# Continued twitching of the latissimus dorsi miniflap after breast conservation therapy: a case report

**DOI:** 10.1186/1477-7819-10-122

**Published:** 2012-06-28

**Authors:** Du-Ping Huang, Xiao-He Ye, You-qun Xiang, Xiao-Hua Zhang

**Affiliations:** 1 Department of Oncology, The First Affiliated Hospital of Wenzhou Medical College, Wenzhou, 325000, China; 2 Department of Radiology, The First Affiliated Hospital of Wenzhou Medical College, WenZhou, 325000, China

**Keywords:** Breast cancer, Breast conservation therapy, Muscle twitching

## Abstract

We report a case of continued twitching of the latissimus dorsi muscle following breast conservation therapy, along with immediate reconstruction with a latissimus dorsi miniflap, which continued despite several attempts at control including BTX-A percutaneous local injection, and was finally cured by delayed division of the thoracodorsal nerve via a small well-tolerated axillary incision.

## Background

A 46-year-old woman was admitted with the complaint of a left breast lump that was noticed 10 days previously. On physical examination, there was a palpable mass measuring about 2.0 cm in the upper inner quadrant of the left breast. Axillary lymph nodes were not palpable. A breast conservation therapy, along with immediate reconstruction with a latissimus dorsi miniflap was performed on the left side in Oct 2005. Tumor excision, axillary nodal clearance, and latissimus dorsi miniflap reconstruction were performed as a one-stage technique once clear tumor margins were confirmed by frozen section. Subsequent paraffin section also confirmed tumor-free margins within 7 days of the first operation. The whole operation was done through a single lateral incision, running from the apex of the axilla, just posterior to the anterior axillary fold, along the lateral border of the breast (displacing the breast laterally often produces a ‘fold’ along this border), ending at 3 o’clock (left breast). She received adjuvant radiation and six cycles of adjuvant chemotherapy with cyclophosphamide, epirubicin, and Docetaxel. She made an uneventful recovery and follow-up examinations were conducted regularly.

Eleven months after discharge she noticed twitching of the transferred muscle in its new anterior position, which soon became persistent. The patient with this condition saw a disturbing superolateral movement of the transferred muscle (receptor area), but it was well tolerated and did not interfere with sleep although her husband noticed that it still occurred then. It was worse when the upper arm was extended. As the problem continued she was referred to the neurology clinic. At the neurology clinic, electromyogram studies showed that the abnormal, voluntary muscle twitching was confined to the latissimus dorsi muscle only and did not involve any other adjacent muscles. She was treated with antispasmodics and anti-epileptiform drugs without success, and then botulinum toxin A(BTX-A) infiltration was prescribed with similar lack of success. The latissimus muscle, however, remained active in the new anterior position and this condition became more distressing. In order to control the constant twitching, delayed division of the thoracodorsal nerve via a small well-tolerated axillary incision was suggested and this was carried out in Dec 2009. And there was no residual twitching of the latissimus dorsi muscle postoperatively.

## Discussions

The latissimus dorsi miniflap is a widely accepted choice for breast-conserving surgery [1,2]. The tendon of the lattisimus dorsi was divided to allow the muscle to reach the breast cavity, attached only by its neurovascular bundle. It is essential to completely divide the latissimus dorsi tendon and serratus anterior perforating branch(es) of the thoracodorsal vessels to obtain sufficient mobility of the flap. Since the late 19th century when its use became widespread, many researches have been done to evaluate its results and complications [1]. In the field of conservative breast surgery, however, little information has been available regarding the complications following immediate latissimus dorsi miniflap reconstruction.

The latissimus dorsi muscle is a wide large flat muscle, located in the lower thoracodorsal area and lumbar region. The muscle assists in adduction and internal rotation of the shoulder. The nerve supply is from the C6-7-8 branches of the brachial plexus, which collect to form the thoraco-dorsal nerve running on the undersurface of the latissimus dorsi muscle [3].

Botulinum toxin A is one of eight exotoxins produced by Clostridium botulinum, a Gram-positive, spore-forming anaerobe [4]. It causes functional denervation of the muscle fibers at the neuromuscular junction by inhibiting the release of acetylcholine from nerve terminals. It has been used clinically since the 1980 for the treatment of strabismus, blepharospasm, and hemifacial spasm and post-traumatic limb spasticity among other spastic conditions [5]. Recently, it was reported to be an effective procedure to treat muscle twitching. However, we failed to control the latissimus dorsi flap twitching by BTX-A percutaneous local injection. This was probably because the various range of response and efficacy of the BTX-A may depend on the different muscle trophism or anatomic variation [6].

In our case, the patient developed complete relief of the latissimus muscle twitching in the anterior position immediately after surgery, indicating the division of the thoracodorsal nerve at a second stage is a straightforward and successful treatment. However, nerve division often leads to atrophy of the muscle; the procedure is only reserved for the small number of cases where spontaneous contraction is a continued problem. We are aware of no other anecdotal report of a similar case, which suggests that this is a rare but mainly unreported phenomenon following breast conservation therapy along with immediate reconstruction with a latissimus dorsi miniflap.

## Conclusions

Continued twitching of the latissimus dorsi muscle is a rare complication of the breast conservation therapy along with immediate reconstruction with a latissimus dorsi miniflap.

## Consent

Written informed consent was obtained from the patient for publication of this case report. A copy of the written consent is available for review by the Editor-in-Chief of this journal.

## Competing interests

The authors declare that they have no competing interests.

## Authors’ contributions

XYQ, XHZh, and XHY analyzed and collected the patient data. HDP was a major contributor in writing the manuscript. All authors read and approved the final manuscript.
